# Multiple sclerosis diagnostic delay and its associated factors in Upper Egyptian patients

**DOI:** 10.1038/s41598-023-28864-x

**Published:** 2023-02-08

**Authors:** Eman M. Khedr, Islam El Malky, Hussein B. Hussein, Doaa M. Mahmoud, Ayman Gamea

**Affiliations:** 1 Neuropsychiatric Department, Faculty of Medicine, Assiut University Hospital, Assiut, Egypt; 2 Neuropsychiatric Department, Faculty of Medicine, Aswan University Hospital, Aswan, Egypt; 3 Neuropsychiatric Department, Faculty of Medicine, South Valley University, Qena University Hospital, Qena, Egypt

**Keywords:** Diseases, Neurology

## Abstract

The earlier the diagnosis of multiple sclerosis (MS), the sooner disease-modifying treatments can be initiated. However, significant delays still occur in developing countries. We aimed to identify factors leading to delayed diagnosis of MS in Upper Egypt. One hundred forty-two patients with remitting relapsing MS (RRMS) were recruited from 3 MS units in Upper Egypt. Detailed demographic and clinical data were collected. Neurological examination and assessment of the Disability Status Scale (EDSS) were performed. The mean age was 33.52 ± 8.96 years with 72.5% of patients were females. The mean time from symptom onset to diagnosis was 18.63 ± 27.87 months and the median was 3 months. Seventy-two patients (50.7%) achieved diagnosis within three months after the first presenting symptom (early diagnosis), while seventy patients (49.3%) had more than three months delay in diagnosis (delayed diagnosis). Patients with a delayed diagnosis frequently presented in the period before 2019 and had a significantly higher rate of initial non-motor presentation, initial non-neurological consultations, prior misdiagnoses, and a higher relapse rate. Another possible factor was delayed MRI acquisition following the initial presentation in sixty-six (46.5%) patients. Multivariable logistic regression analysis demonstrated that earlier presentation, initial non-neurological consultation, and prior misdiagnosis were independent predictors of diagnostic delay. Despite advances in MS management in Egypt, initial non-neurological consultation and previous misdiagnoses are significant factors responsible for delayed diagnosis in Upper Egypt.

## Introduction

Multiple sclerosis (MS) is a chronic central nervous system autoimmune disorder mainly affecting productive young and middle-aged adults^1^. The personal, social and economic sequels are directly related to the consequent inflammation and progressive degeneration along the disease course. Even though no curative treatment for MS has been discovered yet, a breakthrough has been made in the disease-modifying treatments (DMT) industry to alter the disease course^2^. Through an expanding landscape of drugs with different mechanisms of action, the aim of treatment has shifted from merely controlling the disease activity to the prevention of any progression^3,4^. Therefore, the earlier the diagnosis of MS can be made, the sooner the DMTs can be initiated to achieve the treatment goals of timely prevention of disability progression with reduced burdens imposed by the disease nature^5^. However, significant delays are still being noticed before reaching MS diagnosis that could withhold timely DMTs administration^6^. A varied number of factors might cause such delays; this may be disease-related due to the variability of clinical and imaging manifestations, physician-related due to insufficient knowledge of the primary care physicians, or system-related due to the unavailability of diagnostic facilities and specialized centers^7,8^. Since the prevalence of MS is rising in Egypt (25/100,000 was recorded from different centers)^9^, and patients can typically live with MS for almost 40 years^10^, this could be a considerable burden in such a developing country like Egypt particularly the South of it, affecting the quality of life of patients and their families alongside the significant economic consequences on the society^11^. Therefore, a strategy that targets factors causing such delays might have the same importance as developing treatment^12^. Therefore, the current study aimed to identify the factors that play a role in delayed diagnosis of multiple sclerosis in a large sample of patients with multiple sclerosis from Upper Egypt.

### Results

The study included 142 patients with MS with a mean age of 33.52 ± 8.96 years. One hundred and three patients (72.5%) were females, while 39 (27.5%) were males. The mean age at onset was 28.37 ± 8.506 (13–50) years (27.52 ± 8.25 years in females while in males, 30.60 ± 8.88 years). The mean age at diagnosis was 30.12 ± 8.627 (16–50) years. The duration of illness in the studied patients ranged from 3 months up to 20 years, with a mean duration of 5.55 ± 4.08 years and a median of 5 years. Patients had 3 relapses as a median number of relapses during their illness. The most prevalent primary symptoms were motor symptoms in 49 patients (34.5%), followed by sensory symptoms and optic neuritis in 28 patients (19.7%) and 27 patients (19%), respectively.

At the onset of symptoms, 55.6% of the patients initially visited a neurologist, while the remaining initially consulted other specialties like orthopedics and ophthalmologists. Eighty patients (56.3%) were initially misdiagnosed at the first medical consultation, with a median delay from the onset of clinical symptoms to MS diagnosis of three months. The current mean EDSS score was 2.93 ± 1.86 SD with a range of 0.50 to 6.50). When females were compared against males, no statistically significant differences could be found regarding age at onset, age at diagnosis, or time to reach MS diagnosis (U = 0.415). There were also no differences between groups regarding the first specialty sought, previous misdiagnosis, frequency of relapses or the current EDSS scores. Other demographic and clinical features were summarized in Table 1).

**Table 1:** Relation between diagnostic delay and different demographic and clinical parameters (n = 142).

	Total number of patients	Early diagnosis (≤ 3 months) (n = 72)	Delayed diagnosis (> 3 months) (n = 70)	*p*-value
Age at onset, Mean ± SD (Range)	28.37 ± 8.506 (13–50)	29.81 ± 8.985 (16–50)	26.89 ± 7.773 (13–47)	0.041*
Age at diagnosis, Mean ± SD (Range)	30.12 ± 8.627 (16–50)	29.93 ± 9.129 (16–50)	30.31 ± 8.141 (16–49)	0.691
Duration of illness in years	63.48 ± 47.76 (3–240)	4.0243 ± 3.27285	6.5821 ± 4.23253	< 0.0001*
Time to diagnosis in months	18.63 ± 27.873 (0–120)	1.2 ± 4.864	36.53 ± 30.758	< 0.0001*
Sex, n (%)				
Male	39 (27.5)	20 (27.8)	19 (27.1)	0.932
Female	103 (72.5)	52 (72.2)	51 (72.9)	
Type of residence				
Urban	74 (52.1)	37 (51.4)	37 (52.9)	0.861
Rural	68 (47.9)	35 (48.6)	33 (47.1)	
Marital status, n (%)				0.747
Single	51 (35.9)	27 (37.5)	24 (34.3)	
Married	87 (61.3)	42 (58.3)	45 (64.3)	
Divorced	3 (2.1)	2 (2.8)	1 (1.4)	
Widow	1 (0.7)	1 (1.4)	0 (0)	
Level of education				0.815
Low-level education	26 (18.30)	11 (15.3)	15 (21.5)	
Basic-level education	69 (48.6)	36 (50.0)	33 (47.1)	
High-level education	47 (33.1)	25 (34.7)	22 (31.4)	
1st presentation, n (%)				
Motor	49 (34.5)	31 (43.1)	18 (25.7)	0.030*
Non-motor	93 (65.5)	41 (56.9)	52 (74.3)	
Optic	27 (19)	12 (16.7)	15 (21.4)	
Sensory	28 (19.7)	10 (13.9)	18 (25.7)	
Fatigue	2 (1.4)	2 (2.8)	0 (0)	
Polyfocal onset	8 (5.6)	2 (2.8)	6 (8.6)	
Vertigo/imbalance	22 (15.5)	11 (15.3)	11 (15.7)	
Diplopia	6 (4.2)	4 (5.6)	2 (2.9)	
1st specialty sought				< 0.001*
Neurologist	79 (55.6)	59 (81.9)	20 (28.6)	
Non-neurologist (ophthalmologist, orthopedic and neurosurgery, general practitioner)	63 (44.4)	13 (18.1)	50 (71.4)	
Misdiagnoses				< 0.001*
Correct diagnosis since the onset of illness	62 (43.7)	60 (83.3)	2 (2.9)	
Prior misdiagnosis	80 (56.3)	12 (16.7)	68 (97.1)	
Current EDSS score, Mean ± SD	3.02 ± 1.91 (0.0–6.5)	2.736 ± 1.85 (0.5–6)	3.314 ± 1.94 (1–6.5)	0.078
Relapse rate	3.04 ± 1.758 (1–10)	2.31 ± 1.47 (1–7)	3.80 ± 1.72 (2–10)	< 0.001*
Time period of first presentation				0.001*
Before 2015	33 (23.2)	10 (13.9)	23 (32.9)	
2015–2018	52 (36.6)	21 (29.2)	31 (44.3)	
2019–2022	75 (40.1)	41 (56.9)	16 (22.9)	

SD, Standard deviation; *p*, *p*-value for comparing between the studied categories.

*Statistically significant at *p* ≤ 0.05.

The overall median diagnostic delay was three months. About seventy-two patients (50.7%) achieved diagnosis within three months after the first presenting symptom, while seventy patients (49.3%) had more than three months delay in diagnosis and treatment initiation. Consequently, patients were divided into two groups; patients who received an early diagnosis of MS (during three months of onset) and those who had delayed diagnosis (after three months of onset).

We found no statistically significant difference between the two groups of patients regarding the different demographic data. However, there was a statistically significant difference between the two groups regarding the age at onset, the initial presentation of whether it’s motor or non-motor, the first specialty sought, previous misdiagnoses, relapse frequency and the year of the first presentation. Patients diagnosed earlier were slightly older than patients with delayed diagnosis (*p* = 0.041). Also, Patients with initial motor presentation were diagnosed earlier than patients with non-motor presentation (2 months versus 6 months, *p* = 0.030). The direct presentation to neurologists in 73.5% of patients who had motor symptoms at onset could explain why those patients acquired MS diagnosis earlier without prior misdiagnosis in 55.1%. Other differences between the two groups of patients with motor and non-motor onset were illustrated in Table 2. Early diagnosis of MS within the first 3 months was achieved in 81.9% of patients who presented initially to a neurologist compared to only 18.1% of patients who presented to other medical specialties (ophthalmologists followed by orthopedics were the most common non-neurological specialists visited by 17.6% and 16.9% of patients respectively). At least one prior misdiagnosis was given in about 97.1% of patients with a delayed diagnosis, which could be explained by the low index of suspicion for MS with consequently delayed referral to an MS specialist. The significant differences in the frequency of relapses could be explained as a factor or a consequence of delayed diagnosis. Delayed diagnosis will lead to delayed DMT initiation and therefore increase relapse frequency. Also, misdiagnosing a prior symptom may shift attention from MS suspicion. Although reluctance in seeking medical advice is a well-known factor in developing countries like Egypt; it was found to be independent from socioeconomic or educational level. Also, differences in the distribution of specialized neurology services and imaging facilities between urban and rural areas couldn’t explain such delays. However, the notable improvement found in MS diagnosis over the years, especially from 2019 to date, might reflect the increased awareness among laypersons with improved recognition of the possible neurological symptoms and improved healthcare-seeking behavior. Alongside the improved awareness of MS among healthcare providers and the healthcare system, this could be a possible explanation of the trend towards the shorter time to diagnose MS in more recent years. The multivariable logistic regression analysis model demonstrated in SD: Standard deviation Table 3 confirmed that seeking the advice of a non- neurologist, receiving a prior misdiagnosis, and the earlier year period of presentation were the three independent predictors of MS diagnostic delay.

**Table 2:** Criteria of patients according to the initial presentation (motor Vs non-motor).

	Motor presentation (n = 49)	Non-motor presentation (n = 93)
Age at onset, mean ± SD	30.43 ± 9.501	27.28** ± **7.768
Sex, no (%)		
Males	17 (34.7)	22 (23.7)
Females	32 (65.3)	71 (76.3)
First specialty sought no (%)		
Neurologist	36 (73.5)	43 (46.2)
Ophthalmologist	0	25 (26.9)
Orthopedic	12 (24.5)	12 (12.9)
Neurosurgery	0	3 (3.2)
General practitioner	1 (2.0)	7 (7.5)
ENT	0	3 (3.2)
Previous misdiagnosis no (%)		
None	27 (55.1)	35 (37.6)
Vascular disorder	7 (14.3)	11 (11.8)
Orthopedic condition	14 (28.5)	14 (15.0)
Space occupying lesion	1 (2.0)	0
Ophthalmological disorder	0	18 (19.4)
ENT condition	0	5 (5.4)
Psychiatric condition	0	4 (4.3)
Vitamin D deficiency	0	4 (4.3)
Trigeminal neuralgia	0	2 (2.2)
Time to diagnose MS in months, median	2	6

SD, standard deviation.

**Table 3:** Univariate and multivariate logistic regression analysis for the parameters affecting diagnostic delay in the bivariate analysis (n = 142).

	Univariate		^#^Multivariate	
	*p*	OR (95% CI)	*p*	OR (95% CI)
The time of the first presentation	< 0.001*	0.224 (0.108–0.464)	0.043*	0.255 (0.068–0.956)
Before 2019				
2019–2022®				
Age at onset	0.043*	0.959 (0.922–0.999)	0.131	0.940 (0.868–1.018)
1st presentation	0.031*	2.184 (1.073–4.445)	0.493	1.620 (0.408–6.424)
Motor presentation ®				
Non-motor presentation				
1st specialty sought	< 0.001*	11.346 (5.132–25.086)	0.034*	4.244 (1.115–16.151)
Neurologist ®				
Non-neurologist				
Misdiagnoses	< 0.001*	170.0 (36.566–790.347)		
Correct diagnosis®			< 0.001*	91.697 (17.150–490.279)
Prior misdiagnosis				
Relapse rate	< 0.001*	1.860 (1.431–2.418)	0.054	1.576 (0.992–2.502)

OR, Odd’s ratio; CI, Confidence interval.

*Statistically significant at *p* ≤ 0.05.

^#^All variables with *p* < 0.05 was included in the multivariate.

When radiological data were reviewed for possible factors contributing to the diagnostic delay, sixty-six (46.5%) patients had no MRI exams at their initial presentation for different reasons; mainly because their physicians didn’t ask for or they just had a CT scan instead (in 63 patient), while only 3 patients reported a delay in acquiring MRI for their unaffordable price. For those who had an MRI scan immediately after presentation, 17 (12%) of patients showed atypical or incomplete data to fulfil MS diagnostic criteria and were given an alternative initial diagnosis. The radiological data analysis was not incorporated as a possible factor for the diagnostic delay in the logistic regression model as the initial MRI exams of 45 (31.8%) patients were not available and only follow-up images at later stages of the disease were reviewed. Also, different MRI machines with different resolutions and different protocols used to acquire these images have complicated data analysis and comparison. A summary of the radiological data was provided in Table 4.

**Table 4:** MRI data at initial presentation.

	Total number of patients (n = 142)	Early diagnosis (≤ 3 months) (n = 72)	Delayed diagnosis (> 3 months) (n = 70)	*p*-value
MRI availability after initial presentation				
MRI done initially	77 (45.2)	64 (88.9)	13 (18.6)	< 0.001*
No MRI requested initially	62 (43.7)	6 (8.3)	56 (80.0)	< 0.001*
No MRI due to financial issues	3 (2.1)	2 (2.8)	1 (1.4)	1.000
No MRI due to lack of facilities	0	0	0	–
MRI data of the early images				
Sufficient data with MS diagnosis	125 (88.0)	69 (95.8)	56 (80.0)	0.004*
Non-sufficient MRI data or atypical findings	17 (12.0)	3 (4.2)	14 (20.0)	

*p*, *p*-value for comparing between the studied categories.

*Statistically significant at *p* ≤ 0.05.

## Discussion

This research was conducted to investigate the diagnostic delay in MS and the contributing factors in the upper Egyptian population. Given the rising prevalence and incidence of MS in recent years, as well as the importance of early diagnosis and treatment to reduce patients' disabilities and the imposed economic burden, investigating these factors is critical.

In the present study, the mean age at the onset of MS was 28.37 ± 8.506 years, which is somewhat considered younger compared with most studies from other geographical locations. The age at onset of MS symptoms was reported to be 29.35 and 28.2 years in two different studies from Iran^4,13^ and 33.7 years in Canada^14^. In another study from Spain, the mean age of patients at the onset of the disease was 31.2 years^15^. In Colombia, the mean age at the time of the first symptom was 32.4 ± 10.6 years, and at the time of diagnosis was 34.5 ± 10.5 years^16^. On the other hand, women had a typical representation in our cohort (72.5%), following the known global epidemiology of MS and the MENA area, with a female–to–male ratio of 2.6:1^4^.

The most common initial presenting symptoms were motor symptoms related to the pyramidal system involvement, followed by sensory symptoms and optic nerve involvement. This is also similar to the data obtained from the large registry of the MENACTRIMS (Middle East North Africa Committee for Treatment and Research in Multiple Sclerosis)^9^. In the present study, the mean duration of illness was 5.55 ± 4.04 years, the annual frequency of relapses was 3.35 ± 1.7 and the mean EDSS scores of 2.9 ± 1.86. These results were concordant with the conclusion of the MENACTRIMS study reporting that except for an earlier age at onset and a more aggressive clinical course leading to earlier disability, the clinical phenotype of MS in the MENA region was similar to the Western phenotype^9^.

According to the National Institute of Clinical Excellence (NICE), a timeline of no longer than three months is recommended to diagnose MS (< 6 weeks between onset symptoms and the first neurological consultation and < 6 weeks between the neurological consultation and MS diagnosis)^17^. In this study, the median delay between the onset of symptoms and MS diagnosis was three months. This was a month longer than the median delay of two months reported in a study from Iran by Ghiasian et al.^18^. In Portuguese, the median time between the first presentation and diagnosis was 9 months^19^. In the study by Fernandez et al.^15^, the delay was 24.9 months. In Colombia, the overall mean diagnostic delay was 3.07 years (SD 3.83), and about 45.7% of the patients had a delayed diagnosis^16^. The possible reasons for the observed variations between countries are the different levels of health literacy among different populations, different ways the referral processes work, the strength and accessibility to specialized health care facilities and the use of different diagnostic criteria. Table 5 shows the differences in the time to diagnosis between different studies from different populations.

**Table 5:** Time to diagnosis of MS among Egyptian patients and patients from other countries.

Study	Reference	Sample size	Mean delay in diagnosis (SD)	Median delay in diagnosis (IQR)
Current study		142	18.63 (27.87)	3 (23)
Hamadan, Iran	^18^	351	18.01	2
Iran	^13^	536	7 (14.5)	1 (0–6)
Croatia	^20^	39	8 (–)	2.2 (–)
Spain	^15^	149	24.9 (–)	(–)
Portuguese	^19^	285	(–)	9 (2–38)
Denmark	^21^	8947	47.5 (57.5)	(–)
North America	^22^	8983	84.3 (88.8)	(–)
Ireland	^8^	119	2.4 (–)	1.5

The mean (SD) and median (IQR) were reported by month.

Regarding age, in the current study, we found a statistically significant difference between the two groups regarding the age at onset (*p* = 0.041), where the diagnostic delay was more common at a younger age of onset. This was consistent with a previous Egyptian study from Ain Shams University, which found that those younger at the onset of MS experienced diagnostic delays, and denial of symptoms was a leading cause of delayed time to first medical consultation^23^. Furthermore, studies from Canada, Denmark, and the United States of America found that younger onset patients had significantly longer delays, which could be explained by the fact that younger people seek care less frequently, contributing to the longer time to diagnosis^14,24,25^. In contrast, In Portuguese, older age at onset was associated with a longer time to diagnosis, which was primarily explained by a broader age-related differential diagnosis of MS^12^. In Colombia, no link was found between age at onset and delayed diagnosis^16^. These differences between studies could be also attributed to differences in MS prevalence, differences in MS risk factors leading to the variability of the age at onset in different countries, or even different paths of access to the diagnostic facilities^18^.

When patients were compared based on their gender, no statistically significant difference between males and females regarding the time to reach MS diagnosis (U = 0.415). There were no differences between both groups regarding the first speciality sought, previous misdiagnosis, frequency of relapses or the current EDSS scores. These findings regarding the difference between gender were in line with the previous reports^6,14,16,19,26^. In contrast to the study from Iran, patient delay in seeking medical advice was noted in men compared to women^18^. This was explained by the men’s delayed help-seeking behavior, thus potentially hindering diagnostic processes^27^.

Although it was theoretically hypothesized that the presence of a spouse might shorten the time needed to diagnose by the earlier discerning of symptoms and encouraging early medical consultation, we could not find a statistically significant difference between the mean diagnostic delay and marital status. Not many studies specifically looked at how marital status affects the time to MS diagnosis. Still, one Iranian study found that married patients experienced shorter diagnostic delays than single patients^18^.

Regarding education, no statistically significant difference was found between patients with a low educational level compared to those with higher levels in the mean diagnostic delay, contrasting some other reports from the literature^18^.

From a clinical perspective, the initial symptoms could affect the time to diagnosis, where syndromes typical for MS prompt immediate diagnosis while other symptoms might be vague and lead to various differential diagnoses. In the current study, patients with motor versus non-motor onset had a statistically significant difference in the median diagnostic delay. While In Portuguese, the motor deficits were associated with a long diagnostic delay, as the minority first examined by a neurologist explaining part of this delay^19^. Although most studies reported that onset symptoms were independently associated with diagnostic delay, the exact onset symptom associated with such delays was contrasted in different studies from different countries. For example, optic neuritis was associated with a shorter diagnostic delay in Spain^15^ and Iran^18^, owing to an earlier presentation to neurologists. In contrast to the Canadian and Danish cohorts, optic neuritis was associated with lengthier time to MS diagnosis, which was explained by the rapidly resolving nature of the symptom^14,24^. Contrasting reports on the sensory symptoms at the debut of illness also exist, where in the Canadian cohort^28^ was associated with a shorter time to diagnosis, in contrast to the previous study from Egypt as well as the Iranian cohorts where it was associated with a longer delay explained by being a non-specific presentation that can be linked to several etiologies^13,23^.

Patients can present to doctors of different specialties depending on the nature of their symptoms (e.g., general practitioners, ophthalmologists, orthopedics, or neurosurgeons), and if MS is suspected, a consultation of a neurologist or even MS specialist is needed^29^. Almost half of the patients in the current study were first examined by a non-neurological specialty, a factor that independently predicted the diagnostic delay when compared to patients seen by neurologists (*p* < 0.001). On examining the literature, considerable delays result from delayed contact with an MS expert, which impact treatment options and the chance for early intervention^14^. This finding was supported by an international multicentric study that assessed neurologist's knowledge of MS diagnosis and treatment and found that 27% of neurologists had no or only basic awareness of the 2017 McDonald criteria, and more than one-third of neurologists had no or only basic understanding of atypical MS presentations^30^. Although ophthalmology and orthopedic services were the first specialties consulted in the previous Egyptian study (as in the current study), the most prevalent referral source to a neurologist was family and media recommendations^23^. So, increasing the awareness of MS symptoms among laypeople, primary care practitioners, and general neurologists might reduce the time to MS diagnosis.

In the current study, 56.3% of the patients received a previous misdiagnosis leading to a statistically significant delay in the mean diagnostic time (*p* < 0.001) and was an independent predictor of the diagnostic delay. Almost half of the patients in the Portuguese cohort have also received an incorrect prior diagnosis, which led to a longer delay in MS diagnosis^19^.

As was evident in the current study, the number of patients with a delayed diagnosis has decreased over time, and the time to diagnosis has shortened significantly. Also, presentation in earlier periods was an independent predictor for the diagnostic delay, in concordance with previous findings^13–16^. A possible explanation might be the increased awareness of the neurological symptoms among the public, alongside the updated MS diagnostic criteria over the years, making it easier to confirm the diagnosis earlier. More importantly, the approval and availability of several treatment options supported by either insurance or government funding have increased awareness of MS among neurologists^13^.

Despite the latest advances in MS management and increasing availability of DMTs in Egypt's healthcare insurance and governmental funding systems in the last few years, seeking a non-neurologist at initial presentation and previous misdiagnoses are significant factors to the delayed diagnosis in Upper Egypt.

The most important barrier was the lack of an appropriate case registry, with many patients not remembering important data and dates regarding their disease course. Also missing radiological data at the initial presentation together with the use of different MRI machines without a standard MS protocol have limited further analysis of these data as possible factors for the diagnostic delay. Other factors could be evaluated in further studies, like the costs of the specialist visits, the investigatory tools like MRI, CSF Oligoclonal bands, and electrophysiological studies alongside their availability. Moreover, large-scale nationwide studies are recommended.

## Methods

### Participants

**Figure 1:**
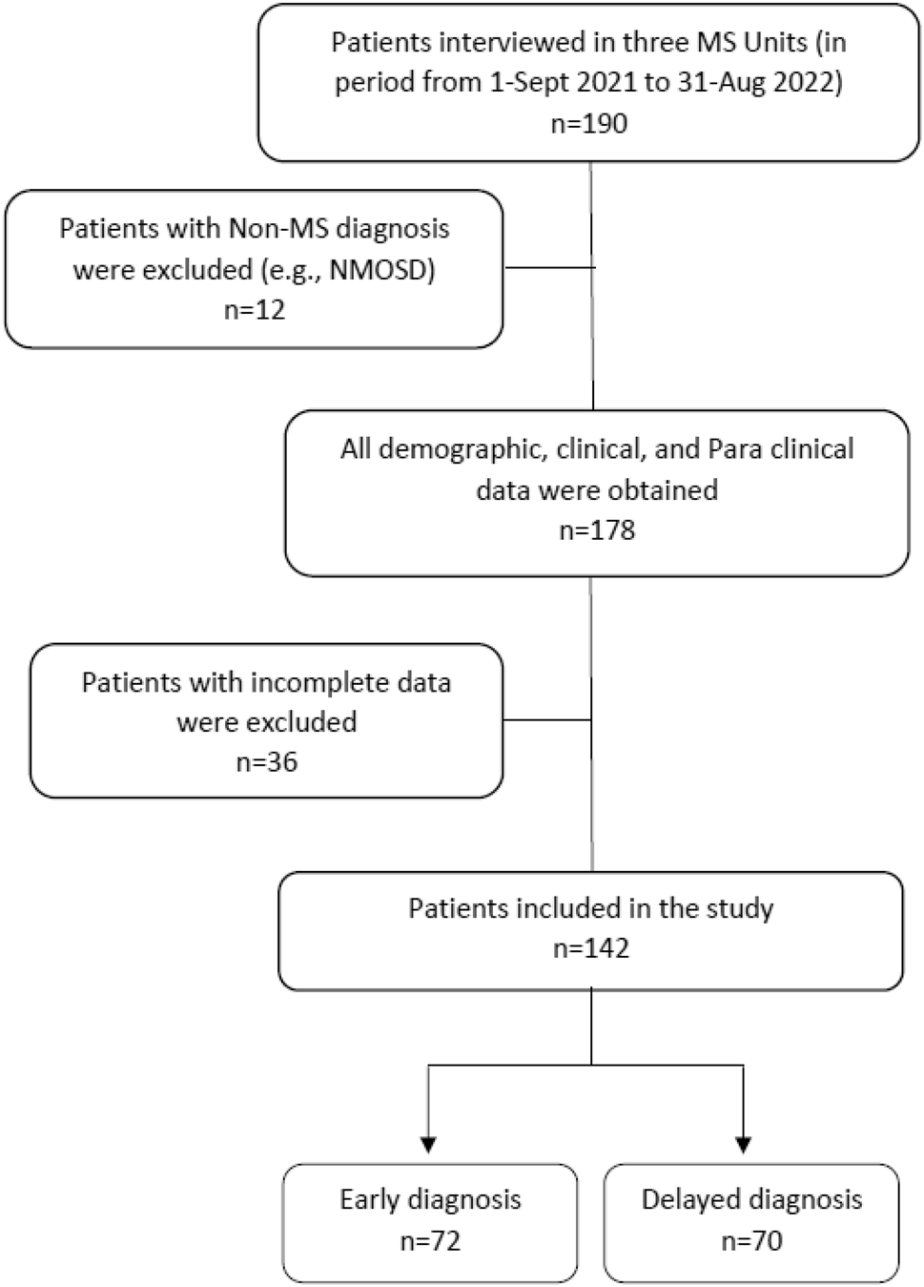
Flowchart of patient selection.

One hundred ninety patients diagnosed with RRMS on DMTs were recruited from three MS centers. Patients were adults of both sexes diagnosed with RRMS according to the 2017 McDonald’s criteria^31^. After excluding patients with any clinical or radiological finding suggestive of demyelinating diseases other than MS, patients with systemic disease or on long-term treatments, patients with incomplete clinical or radiological data, or patients who declined to provide written informed consent, 142 patients were included in the study (Fig. 1).

### Study procedures

This cross-sectional, multicenter, hospital-based observational study included three MS units from three governorates in Upper Egypt (Assiut, Qena, and Luxor). After meeting the inclusion and exclusion criteria, all eligible cases with MS were interviewed and examined by specialized neurologists. Different demographic data, complete clinical data, a complete neurological examination, including the Expanded Disability Status Scale (EDSS) application, and revision of various radiological, electrophysiological, and CSF examinations were collected.

### Ethics

This study was approved by the Local Ethics Committee of Faculty Medicine, South Valley University **(**IRB no: SUV MED NAP020220756**)** and was conducted in accordance with the provisions of the Declaration of Helsinki. Also, written informed consent from all participants was obtained after the description of the aim of the study and methods before participation in the study.

### Statistical analysis of data

The IBM Statistical Package for Social Sciences version 20.0 was used for data analysis (Armonk, NY: IBM Corp). The qualitative variables were described using ratios and percentages. The Shapiro–Wilk test was used to confirm the normality of the distribution. Quantitative variables with normal distributions were reported using mean and SD, while those without normal distributions were described using median and mid-quartiles. The level of statistical significance was set at p 0.05. Diagnostic delay was analysed as a dichotomous variable and classified as an early diagnosis (diagnosed within the first three months) or a delayed diagnosis (diagnosed beyond three months). We chose three months as the time frame suggested by NICE guidelines^17^, after which diagnosis is considered delayed. The delayed diagnosis was the considered outcome variable, and multivariate logistic regression analysis was employed to identify variables that can be regarded as independent predictors of diagnosis delay.

## Data Availability

Data can be made available to qualified investigators upon reasonable request to the corresponding author.

## References

[1] Stenager E (2019). A global perspective on the burden of multiple sclerosis. Lancet Neurol..

[2] Stahnke AM, Holt KM (2018). Ocrelizumab: A new B-cell therapy for relapsing remitting and primary progressive multiple sclerosis. Ann. Pharmacother..

[3] Derfuss T, Mehling M, Papadopoulou A, Bar-Or A, Cohen JA, Kappos L (2020). Advances in oral immunomodulating therapies in relapsing multiple sclerosis. Lancet Neurol..

[4] Moradi N, Sharmin S, Malpas C, Ozakbas S, Shaygannejad V, Terzi M (2021). Utilization of multiple sclerosis therapies in the middle east over a decade: 2009–2018. CNS Drugs.

[5] Damal K, Stoker E, Foley JF (2013). Optimizing therapeutics in the management of patients with multiple sclerosis: A review of drug efficacy, dosing, and mechanisms of action. Biologics Targets Ther..

[6] Giovannoni G, Butzkueven H, Dhib-Jalbut S, Hobart J, Kobelt G, Pepper G, Sormani MP, Thalheim C, Traboulsee A, Vollmer T (2016). Brain health: Time matters in multiple sclerosis. Multiple Sclerosis Relat. Disord..

[7] Gaitán MI, Correale J (2019). Multiple sclerosis misdiagnosis: A persistent problem to solve. Front. Neurol..

[8] Kelly SB, Chaila E, Kinsella K, Duggan M, McGuigan C, Tubridy N, Hutchinson M (2011). Multiple sclerosis, from referral to confirmed diagnosis: An audit of clinical practice. Mult. Scler. J..

[9] Yamout BI, Assaad W, Tamim H, Mrabet S, Goueider R (2020). Epidemiology and phenotypes of multiple sclerosis in the Middle East North Africa (MENA) region. Multiple Sclerosis J. Exp. Transl. Clin..

[10] Scalfari A, Knappertz V, Cutter G, Goodin DS, Ashton R, Ebers GC (2013). Mortality in patients with multiple sclerosis. Neurology.

[11] Ziemssen T, Derfuss T, de Stefano N, Giovannoni G, Palavra F, Tomic D, Vollmer T, Schippling S (2016). Optimizing treatment success in multiple sclerosis. J. Neurol..

[12] Waubant E (2012). Improving outcomes in multiple sclerosis through early diagnosis and effective management. Prim. Care Companion CNS Disord..

[13] Mobasheri F, Jaberi AR, Hasanzadeh J, Fararouei M (2020). Multiple sclerosis diagnosis delay and its associated factors among Iranian patients. Clin. Neurol. Neurosurg..

[14] Kingwell E, Leung AL, Roger E, Duquette P, Rieckmann P, Tremlett H (2010). Factors associated with delay to medical recognition in two Canadian multiple sclerosis cohorts. J. Neurol. Sci..

[15] Fernández O, Fernández V, Arbizu T, Izquierdo G, Bosca I, Arroyo R (2022). Characteristics of multiple sclerosis at onset and delay of diagnosis and treatment in Spain (The Novo Study). J. Neurol..

[16] Cárdenas-Robledo S, Lopez-Reyes L, Arenas-Vargas LE, Carvajal-Parra MS, Guío-Sánchez C (2021). Delayed diagnosis of multiple sclerosis in a low prevalence country. Neurol. Res..

[17] National Institute for Health and Clinical Excellence Guidelines, 2004. http://www.nice.org.uk/nicemedia/live/10930/46699/46699.pdf

[18] Ghiasian M, Faryadras M, Mansour M, Khanlarzadeh E, Mazaheri S (2021). Assessment of delayed diagnosis and treatment in multiple sclerosis patients during 1990–2016. Acta Neurol. Belg..

[19] Aires A, Barros A, Machado C, Fitas D, Cação G, Pedrosa R, Cerqueira J, Perdigão S, da Silva AM, Vale J, Sá MJ, Andrade C, Cacao G, Pedrosa R, Cação G, Pedrosa R, Cerqueira J, Perdigão S, Andrade C (2019). Diagnostic delay of multiple sclerosis in a portuguese population. Acta Med. Port..

[20] Adamec I, Barun B, Gabelić T, Zadro I, Habek M (2013). Delay in the diagnosis of multiple sclerosis in Croatia. Clin. Neurol. Neurosurg..

[21] Thormann A, Sørensen PS, Koch-Henriksen N, Laursen B, Magyari M, Sorensen PS, Koch-Henriksen N, Laursen B, Magyari M (2017). Comorbidity in multiple sclerosis is associated with diagnostic delays and increased mortality. Neurology.

[22] Marrie RAA, Horwitz R, Cutter G, Tyry T, Campagnolo D, Vollmer T (2009). Comorbidity delays diagnosis and increases disability at diagnosis in MS. Neurology.

[23] Tohamy AA, Swelam MS, Abdelgawad DM, Aref HA (2020). Causes of delayed diagnosis of multiple sclerosis in egypt. QJM Int. J. Med..

[24] Esbjerg S, Keiding N, Koch-Henriksen N (1999). Reporting delay and corrected incidence of multiple sclerosis. Stat. Med..

[25] Marrie RA, Cutter G, Tyry T, Hadjimichael O, Campagnolo D, Vollmer T (2005). Changes in the ascertainment of multiple sclerosis. Neurology.

[26] Kaufmann M, Kuhle J, Puhan MAMA, Kamm CPCP, Chan A, Salmen A (2018). Factors associated with time from first-symptoms to diagnosis and treatment initiation of multiple sclerosis in Switzerland. Mult. Scler. J. Exp. Transl. Clin..

[27] Eccles A (2019). Debate and analysis delayed diagnosis of multiple sclerosis in males: may account for and dispel common understandings of different MS “types”. Br. J. Gen. Pract..

[28] Kaisey M, Solomon AJ, Luu M, Giesser BS, Sicotte NL (2019). Incidence of multiple sclerosis misdiagnosis in referrals to two academic centers. Mult. Scler. Relat. Disord..

[29] Brownlee WJ, Hardy TA, Fazekas F, Miller DH (2017). Diagnosis of multiple sclerosis: progress and challenges. Lancet.

[30] Péloquin S, Schmierer K, Leist TP, Oh J, Murray S, Lazure P (2021). Challenges in multiple sclerosis care: Results from an international mixed-methods study. Mult. Scler. Relat. Disord..

[31] McNicholas N, Hutchinson M, McGuigan C, Chataway J (2018). 2017 McDonald diagnostic criteria: A review of the evidence. Multiple Sclerosis Relat. Disord..

